# Positive association between severity of COVID-19 infection and liver damage: a systematic review and meta-analysis 

**Published:** 2020

**Authors:** Hajar Shokri Afra, Nasrin Amiri-Dashatan, Fatemeh Ghorbani, Iradj Maleki, Mostafa Rezaei-Tavirani

**Affiliations:** 1 *Gut and Liver Research Center, Non-communicable Diseases Institute, Mazandaran University of Medical Sciences, Sari, Iran*; 2 *Proteomics Research Center, Faculty of Paramedical Sciences, Shahid Beheshti University of Medical Sciences, Tehran, Iran*; 3 *Department of Clinical Biochemistry, Faculty of Medicine, Tehran University of Medical Sciences, Tehran, Iran*; # contributed equally as first author

**Keywords:** COVID-19, Coronavirus, Liver damage, Liver enzymes, Meta-analysis

## Abstract

**Aim:**

The current study aimed to report a pooled analysis of the association of the circulating levels of liver enzymes and total bilirubin with severe and non-severe COVID-19.

**Background:**

The ongoing coronavirus outbreak is an important threat to health worldwide. Epidemiological data representing greater risk of liver failure in patients infected with Severe Acute Respiratory Syndrome Coronavirus-2 (SARS-CoV-2).

**Methods:**

Electronic databases were comprehensively searched using Medline, ISI Web of Science, EMBASE, and the Cochrane Library up to July 2020. Outcomes from each relevant study were pooled using a random-effects model. Heterogeneity was analyzed by Q test and I^2^ statistics. Sensitivity analysis was also evaluated.

**Results:**

A total of 24 studies were included (4,246 patients) in this study. We found a significant association of COVID-19 severity with increased levels of ALT [SMD: 1.40 U/L; 95% CI (0.93, 1.88); *P *< 0.05, I^2 ^= 96.5%, *P*_Heterogenity = _0.000 ], AST [SMD: 2.11 U/L; 95% CI (1.40, 2.83); *P *< 0.05, I^2 ^= 97.9%, *P*_Heterogenity = _0.000], LDH [SMD: 3.88 U/L; 95% CI (2.70, 5); *P *< 0.05, I^2 ^= 98.7%, *P*_Heterogenity = _0.000] and TBil [SMD: 1.08 mol/L; 95% CI (0.44, 1.72); *P* = 0.001, I^2 ^= 97.7, *P*_Heterogenity = _0.000], whereas, ALP values [SMD: 0.31; 95% CI (-1.57, 2.20); *P *= 0.74] was not significant between severe and non-severe COVID-19 patients. Moreover, elevated liver enzymes were found more in males [OR: 1.52, (95% CI 1.26, 1.83), *P *< 0.05] with severe COVID-19 infection than in females.

**Conclusion:**

The alterations of liver function indexes caused by SARS-CoV-2 infection suggested a potential prognosis biomarker for screening of severe patients at early stages of the disease.

## Introduction

 The coronavirus disease 2019 (COVID-19) pandemic is defined by the World Health Organization (WHO) as a global health crisis and has challenged the world as if facing another world war (1). Since December 2019 when the Severe Acute Respiratory Syndrome Coronavirus 2 (SARS-CoV-2) first appeared in Wuhan, China (1), it quickly spread around the world, and up to July, 2020, a total of > 20,000,000 confirmed cases and >700,000 deaths have been documented globally in 213 countries across 5 continents (2).

The very diverse clinical features of patients with COVID-19 have been described by several cohort studies and case reports, the most serious one being pneumonia (3-7). Severe disease death is possible due to extensive alveolar damage and progressive respiratory failure (4, 8). A number of complications may occur during the disease progression, especially in critically ill patients hospitalized in intensive care units (ICU), including acute cardiac injury, coagulation disorders, acute kidney damage shock, sepsis, and even multiple organ dysfunction (3, 5, 9). Current COVID-19 studies have reported the liver damage of SARS-CoV-2 upon patient presentation, indicating abnormal levels of aminotransferase (ALT/AST), Lactate dehydrogenase (LDH), Alkaline Phosphatase (ALP), Gamma-glutamyl transferase (GGT), Total bilirubin (TBil) and decreased level of albumin. Higher rates of liver function disturbances seem to associate with the severity of COVID-19 (9-11). The pathological features of liver biopsy specimens from a dead patient with COVID-19 showed that injury to the liver was caused by SARS-CoV-2 infection (12). SARS-CoV-2 greatly resembling SARS-CoV enters the lung cells and cholangiocytes through angiotensin-converting enzyme 2 (ACE2) receptors, the probable mechanism of SARS-CoV-2-related liver damage, then clinical symptoms and manifestations begin to emerge (13). Liver damage may develop by inflammation due to an activated immune system (cytokine storm), pneumonia-mediated hypoxia, or even as a result of drug toxicity in patients at late stages of COVID-19 (13, 14). To provide an up-to-date meta-analysis as a reliable reference for further clinical practice, we analyzed liver function changes and evaluated its association with disease progression in COVID-19 patients. Such data may be applicable for patient management, or lead to identify new treatment opportunities. 

## Methods

This systematic review and meta-analysis was performed based on the STROBE statement (Strengthening the Reporting of Observational Studies in Epidemiology) (15, 16) to assure data integration and dependability of the conclusions. 

**Database resource and search strategy**

 We searched the electronic databases including Medline, ISI Web of Science, EMBASE, Cochrane Library, and Scopus up to July 2020 to collect the literature on this topic. The following MeSH (Medical Subject Headings) terms and keywords were used: (“Liver Function Test”, “Liver Injury”, “ Liver Dysfunction”, “Liver Failure and COVID-19”, “SARS-CoV-2”, “Severe Acute Respiratory Syndrome Coronavirus”, and “Coronavirus”), (“Aspartate aminotransferase”, “AST and COVID-19”, “SARS-CoV-2”, “Severe Acute Respiratory Syndrome Coronavirus”, and “Coronavirus”), (“Alanine aminotransferase”, “ALT and COVID-19”, “SARS-CoV-2”, “Severe Acute Respiratory Syndrome Coronavirus”, and “Coronavirus”), (“Alkaline phosphatase”, “ALP and COVID-19”, “SARS-CoV-2”, “Severe Acute Respiratory Syndrome Coronavirus”, and “Coronavirus”), (“Total bilirubin”, “TBil and COVID-19”, “SARS-CoV-2”, “Severe Acute Respiratory Syndrome Coronavirus”, and “Coronavirus”), and (“Lactate dehydrogenase”, “LDH and COVID-19”, “SARS-CoV-2”, “Severe Acute Respiratory Syndrome Coronavirus”, and “Coronavirus”). In addition, the reference lists, gray literature, conference abstracts, and cited papers of full articles were also manually reviewed. Searches were not limited to the English language. Ethical approval and informed consent will not be applied for because of the relevant data we extracted does not involve any private individual or animal.

**Eligibility criteria of studies selection**

Records were included in this meta-analysis if they were full and observational studies withretrospective design that focused on COVID-19 patients (severe and non-severe), severe cases characterized by hospitalized patients in ICU, and non-severe cases; people with milder symptoms of the disease that were hospitalized in the general ward of the hospital, and studies reporting a description of liver function in patients with coronavirus. Conﬁrmed cases of COVID-19 were deﬁned and diagnosed based on real-time reverse transcriptase–polymerase chain reaction (RT-PCR), chest CT findings, and/or compatible symptoms. Then, duplicate papers were removed across the various studies. Exclusion criteria were as follows: Expert opinion and review articles, studies that used other variables, studies that did not address changes in each of the associated outcomes, and inadequacy information on liver enzymes values in terms of severe and non-severe COVID-19 patients and by contacting the first author or corresponding author if necessary. Literature searching and selection was undertaken independently by three researchers (N. Amiri-Dashatan, M. Koushki, and H. Shokri Afra). All three investigators were trained to find eligibility criteria. Finally, the screened full-text articles of each researcher were compared. 

**Outcomes**

Primary outcomes included were serum levels of ALT, AST, LDH, ALP and TBil in patients with severe and mild (non-severe) forms of COVID-19 infection. Secondary outcomes consisted of sex (male, female) and drug-related treatment (antiviral and antibiotic). 

**Data extraction and** **quality assessment**

Selected articles were reviewed by two investigators and extracted data from eligible articles are listed as follows: First author, publication year, country of study, sample size, mean (SD) age, gender type (male/female), and liver function tests including ALT (U/L), AST (U/L), LDH (U/L), and TBil (mol/L) in all severe and non-severe groups. However, the topic of circulating levels of liver enzymes from each study was extracted with the exception of Ruan’s study due to unreliability and invalidity of the data (36). The quality assessment of selected studies and each included evidence was performed independently by two reviewers using the Newcastle-Ottawa Scale (NOS) (17). The studies were assessed across 4 domains: 1) study population selection, 2) exposure, 3) comparability, and 4) outcome. The maximum score for a study was 9 points. The studies were classified into two categories based on scoring: I) low quality (0 to 4 points), and II) high quality (5 to 9 points). The reviewers resolved any disagreements in scoring by discussion.

**Statistical analysis**

The building of continuous data forest plots and differences in assessment of ALT, AST, LDH, ALP, and TBil levels was performed using standardized mean differences (SMD) and 95% confidence intervals (95%CI). In addition, in this meta-analysis the ORs and 95% CI were considered effect size, with risk of severity of COVID-19, to estimate sex (male, female) and drug-related treatment (antiviral drugs and antibiotics). For each group of variables, a random-effects model was used to calculate severity of COVID-19 infection by weighted average of the SMD and the ORs. Standard deviations (SDs) of the mean difference were calculated using represented methods as follows: First, in reporting studies SEM, SD was estimated using the following formula: SD = SEM × sqrt (n), where n is the number of subjects. Second, when the data are not normally distributed, the SD was calculated using range 4 and range 6. As for small to moderate sample sizes (15 < n ≤ 70) the formula rang 4 is the best calculator for the SD, and for larger sample sizes (n > 70) formula rang 6 provides the best calculator for the SD; where b-a is IQR3-IQR1 (18). The heterogeneity among studies was estimated by the Q test (significance level at p < 0·10), and the I^2^ statistics. The I^2^ statistics are characterized by the percentage of total variation in effect size that can be associated with heterogeneity. Values > 50% or 70% were considered moderate to high heterogeneity, respectively. Sensitivity analysis was calculated using the leave-one-out method; sequentially excluding one study in each turn to evaluate the robustness of the results. The Begg’s rank correlation test and the Egger’s regression asymmetry test was also applied to evaluate the potential publication bias obtained by the funnel plot (19, 20). The CMA (comprehensive meta-analysis) V2 software (Biostat, NJ, USA) (21) was used for this meta-analysis. 𝑃 < 0.05 was considered statistically significant.

## Results

**Flow of literature search**

**Figure 1:**
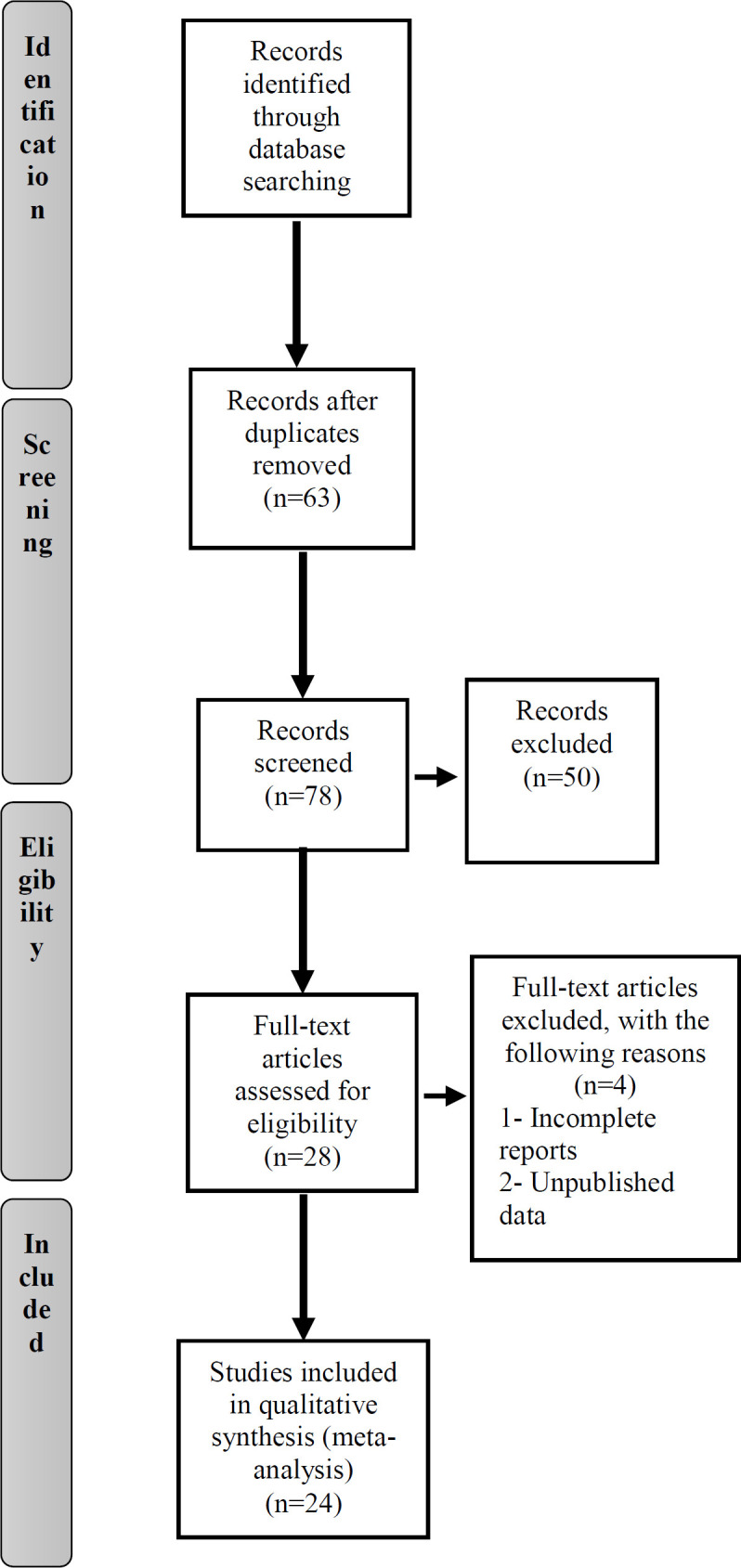
Flow chart of the number of studies identified and proper in the meta-analysis

Our literatures search procedure from electronic databases is presented in Figure 1. In total, 141 records were retrieved using the search strategy, including 138 English and 3 Chinese articles. Of these, 63 records were excluded from our analysis due to duplication. Of the remaining 78 articles, 50 articles were rejected due to lack of enough needed information. Finally, after removal of 4 articles with incomplete reports and unpublished data, a total of 24 eligible records were included in this systematic review and meta-analysis. 

**Fundamental characteristics of eligible studies**

The baseline characteristics of included articles are summarized in Table 1. A total of 4,246 confirmed COVID-19 patients evaluated in our meta-analysis were divided into severe (1,635 patients) and non-severe (2,611 patients) phenotypes. In addition, all included 2020 China published articles (100%) were observational studies in design.

Two studies (8.3%) reported all ALT, AST, ALP, LDH and TBil serum levels, 8 studies (33.3%) had ALT, AST, LDH, and TBil evaluation, and 2 studies (8.3%) only presented TBil levels. According to the NOS quality evaluation, 2 articles were considered low quality and 22 were considered moderate to high quality. The quality assessment results are also presented in Table 1.

**Quantitative data synthesis**

**Figure 2:**
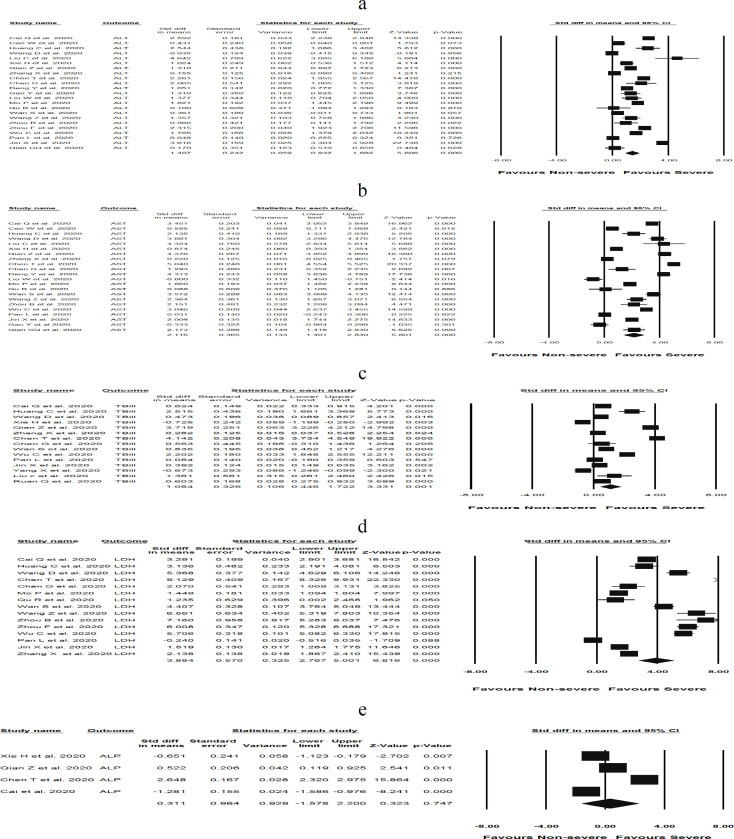
Forest plots assessing standardized mean difference (SMD) and 95% confidence intervals for the association between circulating levels of a) ALT, b) AST, c) LDH, and d) ALP with severity of COVID-19 in admitted and hospitalized patients. Meta-analysis was performed using a random-effects model with inverse variance weighting

**Figure 3:**
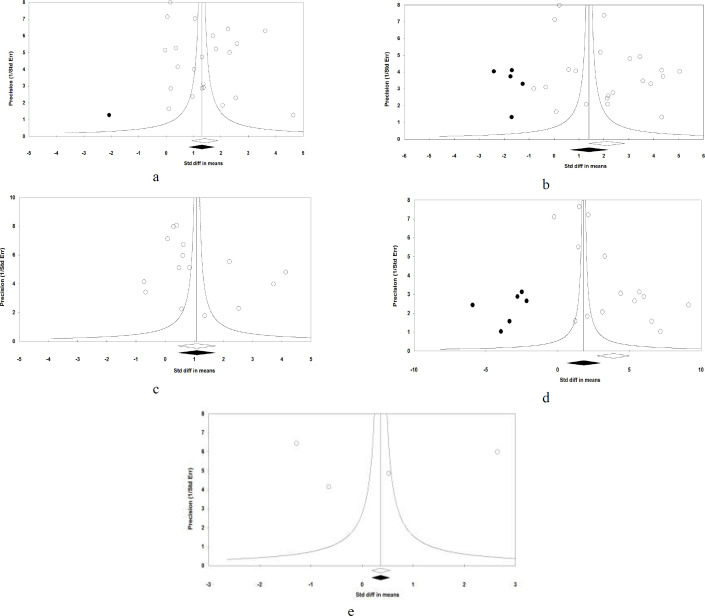
a) Forest plot assessing standardized mean difference (SMD) and 95% confidence intervals for the association between TBil concentration with severity of COVID-19 in admitted and hospitalized patients, and b) Random-effects funnel plot detailing publication bias after trimming and filing in the studies investigating the association between TBil concentration with severity of n-COVID-19 in admitted and hospitalized patients. Open circles represent observed published studies; closed circles represent imputed unpublished studies. Meta-analysis was performed using a random-effects model with inverse variance weighting

We examined liver damage by the assessment of circulating levels of ALT, AST, TBil, LDH, and ALP in COVID-19 patients with severe disease compared to patients with non-severe disease. Remarkably, pooled analyses significantly demonstrated higher levels of ALT [(SMD: 1.40 U/L; 95% CI 0.93, 1.88); *P *< 0.05), I^2 ^= 96.5%, *P*_Heterogenity = _0.000 ] (Figure 2a), AST [(SMD: 2.11 U/L; 95% CI (1.40, 2.83); *P *< 0.05), I^2 ^= 97.9%, *P*_Heterogenity = _0.000] (Figure 2b), and LDH [(SMD: 3.88 U/L; 95% CI (2.70, 5); *P *< 0.05), I^2 ^= 98.7%, *P*_Heterogenity = _0.000] (Figure 2c) in patients with severe COVID-19 compared to non-severe COVID-19. The severity of COVID-19 had no significant effect on the levels of ALP [(SMD: 0.31; 95% CI (-1.57, 2.20); *P *= 0.74), I^2 ^= 99.05%, *P*_Heterogenity = _0.000] (Figure 2d) compared to those with milder patterns. In addition, TBil concentrations [(SMD: 1.08 mol/L; 95% CI (0.44, 1.72); *P* = 0.001), I^2 ^= 97.7, *P*_Heterogenity = _0.000] significantly increased in severe COVID-19 infections compared to non-severe forms (Figure 3a). A statistically significant heterogeneity was observed within included studies in the meta-analysis. Sensitivity analysis interestingly showed no changes in the overall estimates after removing each single low quality study. On the other hand, the circulating levels of ALT [(SMD: 1.28 U/L; 95% CI 0.88, 1.69); *P *< 0.05), I^2 ^= 94.8%, *P*_Heterogenity = _0.000], AST [(SMD: 2.12 U/L; 95% CI 1.33, 2.98); *P *< 0.05), I^2 ^= 98.08%, *P*_Heterogenity = _0.000 ], LDH [(SMD: 4.06 U/L; 95% CI 2.77, 5.36); *P *< 0.05), I^2 ^= 98.7%, *P*_Heterogenity = _0.000 ], and TBil [(SMD: 1.13 U/L; 95% CI 0.42, 1.84); *P *= 0.002), I^2 ^= 97.5%, *P*_Heterogenity = _0.000 ] remained significantly higher in patients with severe COVID-19 following removal of larger sample sizes in the study, which included nearly 37% of the pooled sample size. 

**Publication bias**

**Figure 4:**
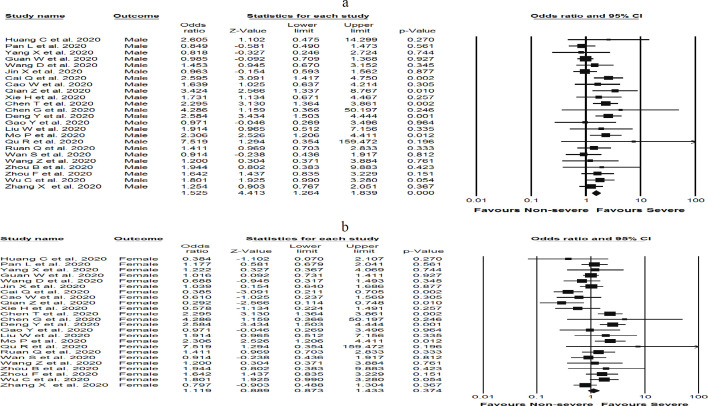
Random-effects funnel plots detailing publication bias after trimming and filing in the studies investigating the association between circulating levels of a) ALT, b) AST, c) LDH, and d) ALP with severity of COVID-19 in admitted and hospitalized patients. Open circles represent observed published studies; closed circles represent imputed unpublished studies

 The Begg’s rank correlation tests (ALT; Kendall’s tau with continuity correction = 0.08, Z = 0.58, 2-tailed *P*-value = 0.56), (AST; Kendall’s tau with continuity correction = 0.00, Z = 0.00, 2-tailed *P-*value = 1), (TBil; Kendall’s tau with continuity correction = 0.24, Z = 1.28, 2-tailed *P-*value = 0.19), (LDH; Kendall’s tau with continuity correction = 0.22, Z = 1.18, 2-tailed *P*-value = 0.23), (ALP; Kendall’s tau with continuity correction = 0.00, Z = 0.00, 2-tailed *P*-value = 1), and the Egger’s linear regression tests (ALT; *P* = 0.60, AST; *P* = 0.18, TBil; *P* = 0.31, LDH; *P* = 0.70, and ALP; *P *= 0.93) were not statistically significant. In addition, the study precision funnel plot (inverse standard error) per effect size (SMD) was symmetric and clearly showed no publication bias in revealing the primary outcomes in patients with severe and non-severe COVID-19 (Figure 3b and Figure 4). 

**Table 1:** Key characteristics of included studies in this systematic review and meta-analysis

Athour's name (year) (Ref)	Country	Samples (N)	Sex N (%)	Mean (SD) Age (year)	Liver Function Test Mean (SD)			Study Quality
					Total	Severe Group	Non-severe Group	
Huang C et al. (2020) (4)	China	Total: 41 Severe: 13 Non-severe: 28	M: 30 (73) F: 11 (27)	49.2 (4.2)	ALT: 33.7 (7.2) AST: 35.5 (3.6) LDH: 305.5 (41.5) TBil: 11700 (1100)	ALT: 60.5 (21.5) AST: 47 (10) LDH: 425.2 (63.7) TBil: 18.2 (5.2)	ALT: 28.3 (5.1) AST: 33.1 (4.1) LDH: 288 (31) TBil: 10.8 (0.7)	6
Pan L et al. (2020) (22)	China	Total: 204 Severe: 105 Non-severe: 99	M: 107 (52) F: 97 (48)	54.9 (15.4)	ALT: 35.8 (48.5) AST: 35.6 (59.6) LDH: 318 (210) TBil: 13300 (10200)	ALT: 37 (63.4) AST: 36.5 (81.4) LDH: 291.8 (189) TBil: 13700 (8100)	ALT: 34.6 (25.4) AST: 34.63 (15.5) LDH: 341.9 (227.5) TBil: 12840 (12000)	6
Yang X et al. (2020) (14)	China	Total: 52 Severe: 32 Non-severe: 20	M: 35 (67) F: 17 (33)	59.7 (13.3)	TBil: 32.6 (7.9)	TBil: 13.1 (4.3)	TBil: 19.5 (11.6)	5
Wang D et al. (2020) (9)	China	Total: 138 Severe: 36 Non-severe: 102	M: 75 (54.3) F: 63 (45.7)	55.5 (4.3)	ALT: 26 (4) AST: 34.3 (4.5) LDH: 276.7 (36.8) TBil: 10500 (9000)	ALT: 24 (9.5) AST: 51 (10) LDH: 442 (73.5) TBil: 12800 (2200)	ALT: 24.2 (3.5) AST: 29.2 (2.8) LDH: 221.5 (20) TBil: 9900 (7000)	8
Jin X et al. (2020) (23)	China	Total: 651 Severe: 74 Non-severe:577	M: 37 (50) F: 37 (50)	46.14 (14.1)	ALT: 26.05 (3.8) AST: 29.5 (2.9) LDH: 320.7 (24.5) TBil: 10.2 (1.1)	ALT: 26.05 (3.7) AST: 29.5 (2.9) LDH: 236.1 (24.4) TBil: 10.2 (1.1)	ALT: 15.2 (2.9) AST: 24.9 (2.2) LDH: 211.6 (14.75) TBil: 9.8 (1.01)	7
Liu C et al. (2020) (24)	China	Total: 32 Severe: 4 Non-severe: 28	-	-	ALT: 31.61 (12.3) AST: 25 (3.3) TBil: 16.4 (2.4)	ALT: 57.5 (16) AST: 39.8 (10.8) TBil: 19.32 (3.4)	ALT: 24.7 (5.2) AST: 23.1 (1.9) TBil: 16.01 (2.3)	7
Cai Q et al. (2020) (25)	China	Total: 298 Severe:58 Non-severe:240	M: 145 (48.6) F: 153 (51.3)	47.2 (4.6)	ALP: 21.9 (2.6) AST: 28.2 (2.4) ALP: 59.6 (4.1) LDH: 262.2 (38.5) TBil: 11.5 (1.3)	ALT: 28.6 (5.2) AST: 37.07 (5.07) ALP: 56.3 (4.3) LDH: 397.6 (92.3) TBil: 12.3 (2.5)	ALT: 20.3 (2.5) AST: 26.8 (2.2) ALP: 61.5 (4) LDH: 237.8 (29.8) TBil: 11.3 (1.3)	5
Cao W et al. (2020) (26)	China	Total: 128 Severe: 21 Non-severe: 107	M: 60 (46.9) F: 68 (53.1)	-	ALT: 31.35 (20.3) AST: 30.63 (18.8)	ALT: 43.8 (47.8) AST: 44.1 (36.2)	ALT: 28.8 (31.8) AST: 27.9 (25.8)	6
Qian ZP et al. (2020) (27)	China	Total: 324 Severe: 26 Non-severe: 298	M: 167 (51.5) F: 157 (48.45)	51.25 (12.16)	ALT: 27.9 (20.02) AST: 29.3 (21.02) ALP: 56.9 (18.9) TBil: 9.5 (4.6)	ALT: 26.3 (3.9) AST: 36.2 (6.9) ALP: 59.1 (5.6) TBil: 11.3 (1.6)	ALT: 22.5 (2.8) AST: 23.7 (2.2) ALP: 57.3 (3.2) TBil: 8.3 (0.7)	5
Qian GQ et al. (2020) (28)	China	Total: 91 Severe: 9 Non-severe: 82	M: 37 (40.66) F: 54 (59.34)	48.37 (3.42)	ALT: 19.25 (2.5) AST: 21.75 (1.83)	ALT: 19.95 (3) AST: 26.18 (0.81)	ALT: 19.5 (2.66) AST: 22 (2)	4
Xie H et al. (2020) (29)	China	Total: 79 Severe: 28 Non-severe: 51	M: 44 (55.69) F: 35 (44.3)	58.5 (3)	ALT: 38.3 (8.2) AST: 33.3 (4.5) ALP: 79.3 (6.9) TBil: 13.4 (1.5)	ALT: 40.5 (13.5) AST: 37.5 (7.5) ALP: 75.7 (8.5) TBil: 12.2 (1.8)	ALT: 31.1 (5.6) AST: 31.5 (6.5) ALP: 83.5 (13.5) TBil: 13.8 (2.4)	6
Chen T et al. (2020) (30)	China	Total: 274 Severe: 113 Non-severe: 161	M: 171 (62) F: 103 (38)	59.5 (4.3)	ALT: 24.75 (3.8) AST: 32 (4) ALP: 69.5 (5.3) LDH: 350.8 (43.45) TBil: 9850 (1130)	ALT: 30.25 (4.8) AST: 47 (6) ALP: 82.5 (9.6) LDH: 568.9 (7.4) TBil: 12800 (1210)	ALT: 21.7 (2.86) AST: 25.8 (2.21) ALP: 64 (4.3) LDH: 266.7 (17.03) TBil: 8450 (900)	6
Chen G et al. (2020) (31)	China	Total: 21 Severe: 11 Non-severe: 10	M: 17 (81) F: 4 (14.2)	56.3 (14.3)	ALT: 30 (16.5) AST: 38.2 (24.6) LDH: 408.1 (231) TBil: 9800 (5600)	ALT: 41.4 (14.9) AST: 51 (28.3) LDH: 567.2 (217.1) TBil: 11200 (6400)	ALT: 17.6 (5.8) AST: 24.2 (4.1) LDH: 234.4 (46.7) TBil: 8200 (3800)	7
Deng Y et al. (2020) (32)	China	Total: 225 Severe: 109 Non-severe: 116	M: 124 (55.1) F: 101 (44.88)	55.5 (3)	-	ALT: 23.2 (3.1) AST: 35.5 (3.3)	ALT: 20.1 (2.8) AST: 23.3 (2.3)	6

M: Male; F: Female; ALT: Alanine Aminotransferase; AST: Aspartate Aminotransferase; ALP: Alkaline Phosphatase; LDH: Lactate Dehydrogenase; TBil: Total Bilirubin

When the estimated missing studies were added to the meta-analysis, the observed and adjusted values for the primary outcomes of ALT, TBil and ALP in patients with COVID-19 remained similar. In other words, when the effect size of missing studies was calculated using the trim-and-fill method for the levels of AST in COVID-19, an additional 5 imputed possibly missing studies reduced the summary SMD to 1.40 U/L [(95% CI 0.63, 2.16)]. As reported by “fail safe N”, 7,553 theoretically missing studies were needed to import the *P*- value to higher than 0.05. Moreover, in reporting studies of LDH in patients with severe and non-severe COVID-19, there was no funnel plot asymmetry and trim-and-fill correction imputed 6 potentially missing studies giving rise to a correct effect size of 1.82 U/L (95% CI 0.63, 3.01). The “fail safe N” method showed that 7,098 theoretically missing studies were required to create the non-significant effect. 

**Sex and risk of liver damage in severe COVID-19 patients**

**Figure 5:**
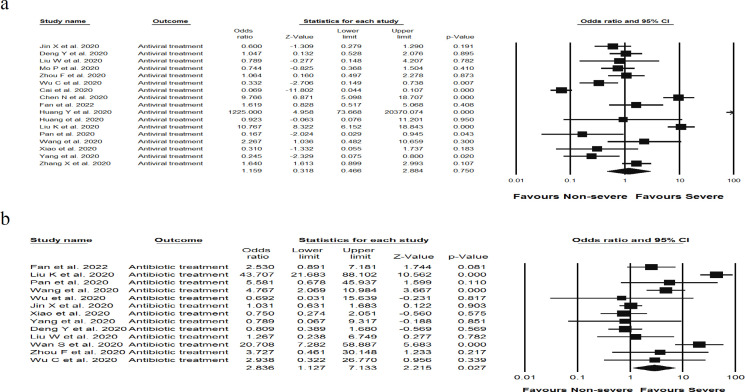
Forest plots detailing Odds Ratio (OR) and 95% confidence intervals between variables of sex, a) male and b) female, and drug treatment, c) antiviral and d) antibiotic drugs, and risk of elevated liver enzymes in admitted and hospitalized patients with COVID-19 infection. Meta-analysis was performed using a random-effects model with inverse variance weighting

Among 24 studies, 1,048 male and 931 female cases with COVID-19 were included in our meta-analysis. The summary OR for males was OR [(1.52), (95% CI 1.26, 1.83), *P *< 0.05, I^2 ^= 33.18%, *P*_Heterogenity = _0.059] compared to females OR [(1.11), (95% CI 0.87, 1.43), *P* = 0.37, I^2 ^= 60.28%, *P*_Heterogenity= _0.000]. Strikingly, our results clearly showed that the levels of liver enzymes in men were significantly higher than in women; both in same-severe status. (Figure 5a and Figure 5b). 

**Drug treatment and risk of liver damage in severe COVID-19 patients**

To determine the association between pharmacological treatment and risk of liver damage, a total of 21 studies were analyzed, including 1,687 severe cases of COVID-19 treated with antiviral drugs, as well as 16 studies containing 915 individuals with severe COVID-19 treated with antibiotics. The results showed that antibiotics-treated patients had a significantly elevated liver enzymes [OR (2.83), (95% CI 1.12, 7.13), *P* = 0.027, I^2 ^= 89.2%, *P*_Heterogenity = _0.000] compared to patients treated with antiviral drugs [OR (1.15), (95% CI 0.46, 2.88), *P* = 0.75, I^2 ^= 94.7%, *P*_Heterogenity = _0.000] (Figure 5c and Figure 5d).

## Discussion

The overall result of this meta-analysis is that the extent of liver damage may strongly depend on the severity of COVID-19 infection. To our knowledge, this is the first study to assess the circulating levels of ALT, AST, LDH, ALP, and TBil, in severe and non-severe hospitalized patients with COVID-19 infection. 

Liver damage, reported in recent published studies, was mainly characterized by liver dysfunction; however, there are contradictory results on liver enzyme values in hospitalized patients with severe and non-severe COVID-19 infection. This meta-analysis demonstrated the incidences of abnormal liver tests in patients with severe COVID-19. The present study showed that the circulating levels of ALT, AST, LDH, and TBil concentrations significantly increased among severe cases compared to milder forms of COVID-19 infection, while the level of ALP had no significant change between severe and non-severe (milder form) COVID-19 patients. Consistent with the present findings, other studies reported the elevated AST, ALT, and total Bilirubin levels (43, 44) correlation with high ranged (> 50%) liver damage in severe COVID-19 patients (44). Moreover, a low increase in ALT and AST levels was attributed to relatively mild (37%) COVID-19-related liver damage (9). As such, combining our results and the findings of previous observational studies collectively suggest that elevated liver enzymes as a prognostic factor associated with COVID-19 severity can effectively predict critical cases in hospitalized patients. The present analysis also showed that sex (male/female) is a key feature for determining COVID-19 severity in hospitalized patients; severe COVID-19 cases were significantly more among males than females. Moreover, we found that liver enzymes were elevated in men more than in females; thus, this finding may support more serious 

COVID-19-related clinical manifestations in males showing that they needed more intensive treatment. In agreement with our results, recently a meta-analysis of observational studies described a significant higher risk of mortality in males than in females (45). Taken together, gender may indeed increase the risk factors of COVID-19 severity due to more liver damage found in males. In clinical and animal models, described in the articles, hepatocytes injury or death was shown to be the trigger for the release of liver enzymes into the circulation. Therefore, liver damage progression can be monitored by their serum levels in patients with liver disease (46). However, in COVID-19 infection, it is still unclear how liver impairment occurs. Studies show that the liver function abnormalities found in patients with SARS-CoV-2 were similar to those found in other types of viral infections. Liver impairment is a common manifestation in SARS-CoV infected patients (47). Moreover, Saad et al. indicated that MERS-CoV-related complications mainly were observed in organs such as the liver and kidneys (48). Hence, three main hypotheses have been proposed including direct virus damage, systemic inflammation, and drug-induced injury. SARS-CoV-2 shares the genetic sequence with SARS-CoV and MERS, and enters the cell with the same receptor, ACE2 (49). It seems SARS-CoV-2 must first enter the blood stream to infect liver tissue. Although the blood viral load (RNAaemia) was detected by the Chen et al (50) and Huang et al. (4) studies, the direct attack of SARS-CoV-2 to hepatocytes, supported by bioinformatics studies, is unlikely due to their low ACE2 expression levels (51). Liver biopsy of patients who died from COVID-19 also confirmed that no obvious intracellular viral inclusions were observed (12) in contrast to large numbers of SARS-CoV particles found in the liver. Nevertheless, pathological features of COVID-19 post-mortem tissue biopsies displayed moderate microvascular steatosis and hepatocyte degeneration, accompanied by mild lobular necrosis suggesting hepatocyte injury, which greatly resemble those seen in SARS and MERS coronavirus infection (12). 

According to high ACE2 expression in cholangiocytes (51), it was then thought that liver damage may be potentially caused by SARS-CoV-2 bile duct infection, not necessarily through the virus entering into hepatocytes. Howsoever, our results showed otherwise; no significant elevation in ALP levels was seen between mild and severe COVID‐19 patients, which may implicate that the SARS-CoV-2 bile duct injury is not very notable. This finding suggested the liver abnormalities in COVID-19 patients may be due to other causes such as systemic inflammation alone or coupled with drug hepatotoxicity. COVID-19 abnormal liver indexes were correlated with the increased levels of inflammation markers including C-reactive protein (CRP), erythrocyte sedimentation rate (ESR), neutrophil-lymphocyte-ratio (NLR), interleukin-2 (IL-2) and IL-6, and tumor necrosis factor TNF(52-54). Furthermore, immune deregulations have significantly occurred in severe COVID-19 cases (55). In fact, systemic inflammation induced by a hyperactivated immune system and cytokine storm related to SARS-CoV-2 infection can affect and damage many organs, especially the liver which is an important organ that maintains physiological immune tolerance to invading pathogens.. Furthermore, hypoxic/Ischemic liver damage was reported in COVID-19 cases with the condition of hypoxia (lung failure) or coupled with hypovolemia (shock) (56). It is worth noting that HDL increase is seen at the early stage of acute liver failure caused by hypoxic/ischemic conditions (55). Our findings also revealed the elevated HDL levels in sever compared to mild COVID-19 cases. Fan et al. reported higher LDH in COVID-19 patients with abnormal liver function than normal ones, and also in COVID-19 patients who expired from respiratory failure (57), which is compatible with the present study findings. Moreover, LDH also increased in SARS and MERS (58, 59), therefore, we speculate that elevated LDH in COVID-19 may have an association with hepatic hypoxic conditions which closely correlate with macrophage over-activation; however, this needs further investigation. Although an elevated level of LDH in COVID‐19 patients can be explained by simultaneous liver and heart involvement because ACE2 is widely expressed in cardiac blood vessels (60). Due to the fact that the liver is the main organ for detoxification, it may be a preferred target of hepatotoxicity in drug treatment. In this regard, we analyzed the risk of drug-mediated liver damage in severe COVID-19 infection. Strikingly, our results indicated significant elevated liver enzymes in antibiotics-treated patients implicating a strong association between injury to the liver and antimicrobial drugs. Recent studies have reported conflicting results, although generally little evidence is currently available on liver function and medications use in COVID-19 infection. Fan et al. observed that the liver function was impaired in severe COVID-19 patients which were treated with Lopinavir/ritonavir antiviral agents (57). Other studies found significant liver damage in severe COVID-19 patients treated with antiviral and antibiotic (41, 42). Ultimately, the present study warns using antibiotics but limited as important potential risk factors which can foster liver damage among patients with severe COVID-19. However, pre-existing liver disease should be considered in COVID-19 patients, which has been ignored in most previous COVID-19 studies. Collectively, despite all these explanations, there is still a great ambiguity in understanding the underlying mechanism of SARS-CoV-2 liver index abnormalities.

This meta-analysis has several limitations: First, we were not able to better assess the COVID-19-induced injury to the liver due to the variation in sample size of included studies, second, the absence of epidemiologic reports from different regions of the world, and finally, large scale clinical trials are needed for more valid results. 

 The data derived from this meta-analysis are significantly valuable as an innovative resource for complete understanding of the relationship between COVID-19 infection and liver damage. In addition, these results might also provide useful information for the discovery of prognosis biomarkers of COVID-19 infection. Nonetheless, further investigations are needed to clarify the exact mechanisms of pathogenesis and confirm the results of this study.
